# Patient in Mayfield Head Pins Requiring Prone Bronchoscopy for Mucus Plug Obstruction: A Case Report

**DOI:** 10.7759/cureus.64270

**Published:** 2024-07-10

**Authors:** Sravya Veligandla, Ioana F Pasca, Trinith Radhakrishnan, Haad Arif, Gian Christian T Ignacio, Ashish Sinha

**Affiliations:** 1 Anesthesiology, Riverside University Health System Medical Center, Moreno Valley, USA; 2 Anesthesiology, Loma Linda University Medical Center, Loma Linda, USA; 3 School of Medicine, University of California Riverside School of Medicine, Riverside, USA

**Keywords:** prone bronchoscopy, chronic obstructive pulmonary disease (copd), posterior cervical fusion, odontoid process fracture, glycopyrrolate, chronic bronchitis, mayfield pins, prone position surgery, mucous plug, flexible bronchoscope

## Abstract

Mucus plug obstruction is a common complication in prone patients associated with loss of ventilation and hemodynamic instability. This case presents a 62-year-old female with chronic bronchitis who underwent posterior cervical fusion for a type III dens fracture with extension into the pars articularis and pedicles. Glycopyrrolate was administered to assist with fiberoptic intubation. After successful intubation, bronchoscopy revealed copious endotracheal secretions requiring preoperative therapeutic removal. Despite extensive removal of thick endotracheal secretions preoperatively, obstructive mucus plugging developed intraoperatively with complete loss of end-tidal carbon dioxide (ETCO_2_) while the patient was in Mayfield head pins. With limited airway access, suctioning and prone flexible bronchoscopy were performed, successfully restoring ETCO_2_. This experience underscores the need for heightened awareness and preparedness for mucus plug obstruction in chronic bronchitis patients undergoing prone cervical spine surgeries.

## Introduction

The prone position poses challenges for the anesthesiologist in regard to addressing intraoperative airway complications. Prone positioning is required in posterior spine procedures, requiring the anesthesiologist to maneuver around a bulky cranial and spine stabilization apparatus which can be a significant obstacle in emergent situations. One possible complication in a prone patient is an obstruction of the endotracheal tube (ETT) by secretions or kinking [1]. Early diagnosis followed by rapid treatment of the potential loss of end-tidal carbon dioxide (ETCO_2_) and ventilation is essential to preventing an impending hypoxic cardiac arrest [1]. Methods of addressing mucus plugs include preoperative suctioning of the airway to remove any sputum and mucus buildup as well as the administration of glycopyrrolate to reduce salivary secretions and albuterol to dilate the airways, respectively [2,3]. The ideal anesthetic management of mucus plugs is anticipating the complication and taking preemptive steps to reduce its incidence [1].

Here, we describe a challenging case of mucus obstruction in a prone patient placed in Mayfield head pins. This manuscript adheres to the applicable EQUATOR guideline. A written Health Insurance Portability and Accountability Act (HIPAA) authorization was obtained from the patient for the publication of this case report.

## Case presentation

**Figure 1:**
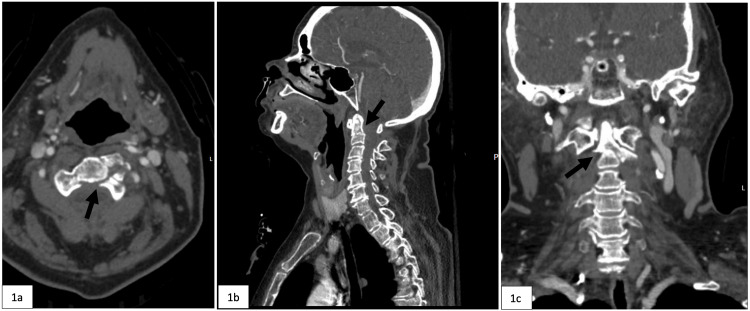
CT images demonstrating the type III dens fracture indicated by the black arrow on (a) axial view, (b) sagittal view, and (c) coronal view CT: Computed tomography

**Figure 2:**
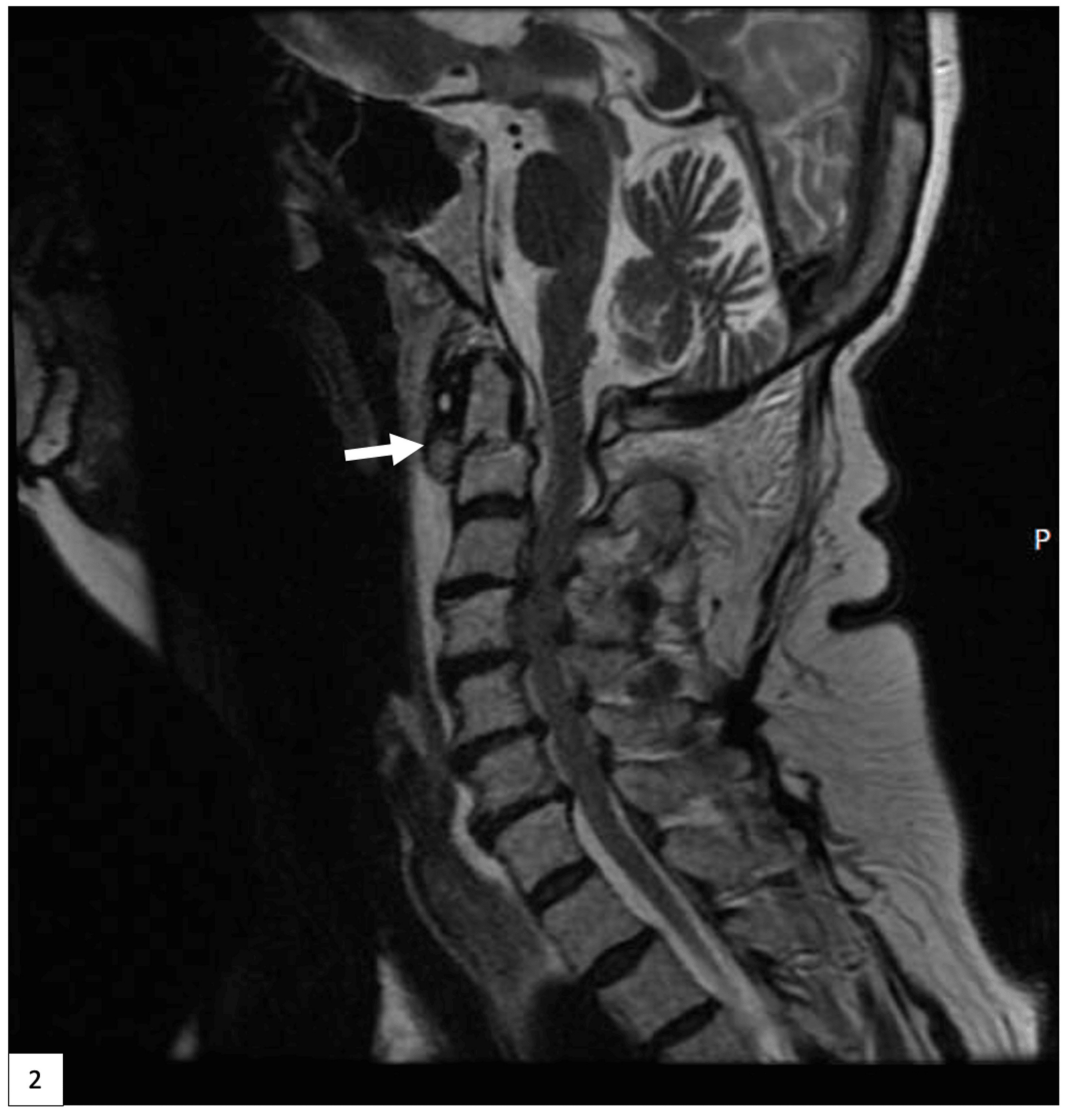
T2 sagittal MRI of the cervical spine demonstrating the type III dens fracture indicated by the white arrow MRI: Magnetic resonance imaging

A 62-year-old female with pertinent medical history consisting of hypertension and chronic bronchitis presented to the emergency department (ED) after a trip and fall at home while intoxicated. Radiographic imaging in the ED revealed numerous facial and cervical spine fractures including fractures to the right maxillary sinus, right orbital floor, possible left condylar neck, as well as a type III dens fracture with extension into the pars articularis and pedicles and a rotatory subluxation of C1 and C2 on the left (Figure 1). Magnetic resonance imaging (MRI) demonstrated disruption of the transverse atlantal ligament and atlanto-occipital membrane as well as severe cervical canal stenosis down to her C6-C7 region (Figure 2). The patient was scheduled for a C1-C4 posterior cervical fusion for her unstable C2 fracture.

Given the patient’s history of chronic bronchitis and productive cough on preoperative evaluation, and the planned surgical approach in the prone position with Mayfield head pins, the decision was made for planned bronchoscopy after intubation. A standard size 7 mm internal diameter (ID) cuffed ETT was inserted aided by fiberoptic and video laryngoscopy without removal of the cervical stabilization collar to prevent movement of the unstable cervical spine. Following intubation, bronchoscopy through the newly inserted ETT revealed thick mucus plugs, concentrated in the right mainstem bronchus. Copious suctioning was performed and all mucus collections in the right and left bronchi were bronchoscopically removed. The patient was then positioned in Mayfield head pins and subsequently into a prone position by the anesthesia and neurosurgery teams.

Approximately four hours after surgical incision, the patient experienced a sudden and complete loss of ETCO_2_ without any discernible movement of the well-taped ETT, and she experienced a decrease in oxygen saturation to the 70s despite attempts at manual breathing bag ventilation. Suctioning was performed on the trachea using a 14 French suction catheter removing a large volume of mucus with no change in the patient's respiratory status. The surgical team was alerted of the patient’s desaturation, the incision was temporarily closed, and an intraoperative chest X-ray was obtained to rule out ETT migration. As the gurney was brought into the room to turn the patient to the supine position, bronchoscopy was performed expeditiously. Extensive mucus plugs were removed from the trachea and bilateral mainstem bronchi, and the patient’s ETCO_2_ and oxygen saturation returned to baseline. The surgical team was then able to complete the operation successfully, without the need to reposition the patient from the prone position.

## Discussion

In this case report, we describe the challenges of addressing mucus plugging in a prone patient while having to maneuver around Mayfield pins. Given the patient’s productive cough during the interview with a history of chronic bronchitis, a condition often associated with airway mucus plugs, we anticipated the possibility of mucus plugging and took preemptive steps to minimize the risk of plugging by using albuterol, glycopyrrolate, and bronchoscopy for removal of secretions [2,3].

The present case underscores the challenges in averting mucus plug formation, even when employing standard preventive practices. Despite these efforts, mucus obstructions persisted, necessitating intraoperative albuterol administration and additional suctioning for effective removal. In 2022, a case report described two patients with loss of ETCO_2_ due to bronchospasm and extensive mucus plugging by the condition "silent lung" in which there is a sudden loss of ventilation and any respiratory sounds via auscultation that ultimately required epinephrine to regain hemodynamic stability [4]. It's worth noting that the use of Mayfield head pins, while a common practice in neurosurgery, poses a challenge when a swift transition to a supine position is required during emergency airway management [5]. In cases where rapid repositioning is not feasible, or where the airway is not easily accessible, applying a flexible bronchoscope is a viable option to visualize and suction the airway. Few case reports and studies exist describing flexible bronchoscopy in the prone position in acute respiratory distress syndrome (ARDS) patients in the intensive care unit (ICU); however, we could find no case reports describing prone bronchoscopy for mucus plug clearance during anesthesia [6]. Two case reports describe prone bronchoscopy during anesthesia: one finding extrinsic tracheal compression during prone spine surgery and another describing two cases of prone bronchoscopy for tracheal narrowing in two patients with Duchenne muscular dystrophy [7,8]. 

Additional methods of improving mucus clearance and preventing mucus obstruction preoperatively include the use of a noninvasive mechanical insufflation-exsufflation (MI-E) device, often referred to as a "cough assist" device, to induce forceful coughing and to clear mucus as well as a preoperative regimen of inhaling nebulized hypertonic saline twice daily [9-11].

Alternative methods of addressing mucus obstruction during surgery includes replacement of the ETT or the removal of the ETT and the use of bag-mask ventilation [1]. This approach requires the patient to be in the supine position, which was not an immediate option in our case due to cervical spine instability. Grimmett et al. discuss the use of an arterial embolectomy catheter to physically remove the obstruction by inflating the balloon distal to the obstruction prior to extracting the catheter [12]. Yet another option is the use of Magill forceps to remove mucus plugs; however, associated limitations include the potential for cuff perforation, risk for mucous membrane damage, and the size of the Magill forceps precluding insertion into the ETT; all of which are difficult in the prone position [13,14]. Mucus plugging in the prone position can occur even after preemptive bronchoscopy in patients with chronic bronchitis and can lead to loss of ETCO_2_, inability to ventilate, and even cardiac arrest. While arrangements are being made to move the patient supine and rescue the airway, prone flexible bronchoscopy with therapeutic removal of secretions may be a life-saving intervention.

## Conclusions

Sudden loss of ETCO_2_, especially in the prone position with Mayfield head pins in place, requires immediate action with potentially catastrophic outcomes if a delay in diagnosis and treatment occurs. Immediate action requires first assessing all monitors and a thorough knowledge of the patient’s perioperative comorbidities. Patients with chronic bronchitis and chronic productive secretions need special attention to decrease the secretion burden and to treat a potential mucus plug should it occur in the prone position. Planning should include having a bronchoscope in the room, carefully taping the ETT to prevent displacement, and discussing with the surgical team in case the patient needs to be turned to supine emergently. Our bronchoscopy in the prone position is one of the first case reports that shows successful removal of the mucus plug without turning the patient supine with immediate return of ETCO_2_ and successful completion of the spine surgery with excellent patient outcome.
